# The biomechanical changes of load distribution with longitudinal tears of meniscal horns on knee joint: a finite element analysis

**DOI:** 10.1186/s13018-019-1255-1

**Published:** 2019-07-25

**Authors:** Kaijia Zhang, Lan Li, Longfei Yang, Jianping Shi, Liya Zhu, Huixin Liang, Xingsong Wang, Xianfeng Yang, Qing Jiang

**Affiliations:** 1 State Key Laboratory of Pharmaceutical Biotechnology, Department of Sports Medicine and Adult Reconstructive Surgery, Drum Tower Hospital affiliated to Medical School of Nanjing University, Nanjing, China; 2 School of Mechanical Engineering, Southeast University, Nanjing, China; 3 School of Electrical and Automation Engineering, Nanjing Normal University, Nanjing, China; 4 School of Mechanical and Electrical Engineering, Nanjing University of Aeronautics and Astronautics, Nanjing, China; 5 Department of Radiology, Drum Tower Hospital affiliated to Medical School of Nanjing University, Nanjing, China; 6 Institute of Medical 3D Printing, Nanjing University, Nanjing, China

**Keywords:** Meniscus, Longitudinal tear, Finite element analysis, Subchondral bone osteonecrosis

## Abstract

**Background:**

Meniscal horns are important structures of meniscus, and longitudinal tears of these places could significantly change the load distribution among the knee joint. Few studies concerned the stress concentrated on bones, which may induce the osteonecrosis of subchondral bone. The goal of this study was to construct a finite element (FE) model with high fidelity of the knee joint and evaluate the biomechanical changes of load distribution of components after longitudinal tears of the horns of meniscus.

**Methods:**

Computed tomography and magnetic resonance images were used to develop the FE model, and two different kinds of simulations, the vertical and the anterior load, mimicking the static stance and slight flexion simulations, were applied after longitudinal tears of the horns of meniscus.

**Results:**

Significantly elevated peak compressive and shear stress was observed on the menisci, cartilages, and subchondral bones, and enlarged meniscus extrusion was noticed. Between all the four types of longitudinal tears investigated in this study, longitudinal tears at the posterior horn of the medial meniscus were found to be the most significant.

**Conclusions:**

These findings showed that longitudinal tears of the meniscal horns lead to increased magnitude and changed distribution of stress and indicated the important role of posterior horn of medial meniscus. This may contribute to the mechanism between meniscal tears and spontaneous subchondral bone osteonecrosis.

**Electronic supplementary material:**

The online version of this article (10.1186/s13018-019-1255-1) contains supplementary material, which is available to authorized users.

## Background

The meniscus is critical for load transmission, stabilization, shock absorption, and lubrication of the knee [1]. It is estimated that the medial and lateral meniscus bear 50% and 70% of the load of the medial and lateral compartment individually [2]. This indicates the essential role in load transmission of the meniscus in the knee joint. Meniscal tears often occur when the knee twists or rotates forcefully, resulting in approximately 650,000 meniscus surgeries annually in the USA [3]. Longitudinal tears run parallel to the peripheral rim of the meniscus and are situated in highly vascularized regions [4, 5]. With regards to all longitudinal tears, tears at the anterior and posterior horn of the meniscus are of the most consequence. The tears can cause abnormal contact between the femur, tibia, and meniscus, resulting in the redistribution of load in the knee joint. The altered load distribution will induce biomechanical changes in the meniscus, cartilage, and even subchondral bone. This will result in the early onset of spontaneous osteonecrosis of the subchondral bone [6]. Hence, it is necessary to examine the effect of meniscal tear on knee biomechanics and altered load redistribution of the meniscus-teared knee.

With respect to knee biomechanics, traditional biomechanical tests using cadaveric joints may reveal specific aspects of the changes of load in the joints [7, 8]. However, with limited specimen resources, the number of test that can be performed are limited and hence will be unable to give a comprehensive picture of all load-bearing compartments in the joint. Hence, finite element analysis is an ideal method to manage these complex issues to accurately analyze the load distribution of the knee joint. In order to attain an ideal finite element model, appropriate geometry and exact mathematical description of the bone, menisci, and ligaments are required. This can provide maximum simulations of the actual knee joint. Previous studies have investigated the influence of meniscal damage on the biomechanical changes of the load redistribution in the knee joint [9, 10]. It has been demonstrated that meniscal tears have a positive impact for the maintenance of high levels of contact stress. This may improve the progression of knee OA [11]. However, these studies focused on the load changing of the meniscus and cartilage and failed to investigate the load of the subchondral bone. Several clinical articles have indicated that the damage to the meniscus could even induce osteonecrosis of the subchondral bone [6, 12, 13] .

The purpose of this study was to develop a detailed finite element model of the knee with the bones, cartilages, meniscus, and main ligaments, so as to investigate the changes to load distribution of the meniscus, cartilage, and subchondral bone after longitudinal tear of the anterior or posterior horn of the meniscus. The results of this study may help explain cartilage and meniscus degeneration and subchondral bone osteonecrosis following tears that occur at the anterior or posterior horn of the meniscus.

## Methods

This study was performed in accordance with all the relevant guidelines and regulations. All experimental study protocols were approved by Drum Tower Hospital, an affiliate of the Medical School of Nanjing University.

### Data acquisition

MR data was obtained from a 35-year-old male using a 3-T clinical MR scanner (uMR 770, United Imaging, Shanghai, P.R.C) and a 12-channel knee send-receive radio frequency coil. The subject was in the supine position and the knee to be examined was positioned in the central region of the coil. A modulated flip angle technique in refocused imaging with extended echo train sequence was performed for each subject using two excitations, 176 continuous slices with a slice thickness of 1.5 mm, repetition time 1000 ms, echo time 56 ms, matrix 240*228, FOV 152 mm, and voxel size 0.67 × 0.63 × 0.64 mm^3^ was used in sagittal planes. The scan time was 6 min and 44 s. The CT data was obtained from the same individual using a GE Lightspeed 16 CT instrument (GE, CT, USA). The scan was performed for the lower limb at the neutral posture with a slice distance of 0.625 mm and a field of view (FOV) of 500 mm.

### 3D reconstruction of the knee joint

**Fig. 1:**
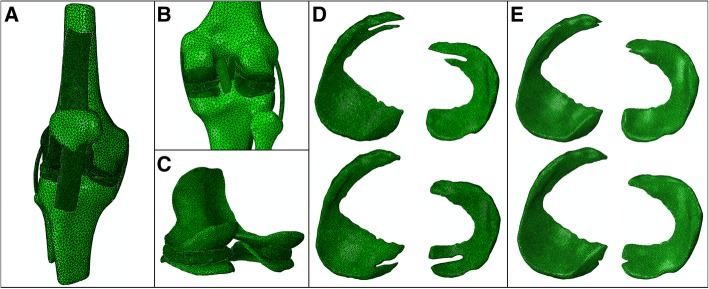
The view of 3D models used in the FE simulation. **a** The general view of knee joint model. **b** The posterior partial enlargement view of knee joint. **c** The rear partial enlargement view of the cartilages and menisci. **d** FE models of longitudinal meniscal tears of anterior and posterior horns. **e** FE models of four smaller longitudinal meniscal tears of anterior and posterior horns

MIMICS 19.0 (Materialise, Leuven, Belgium) was used to reconstruct the 3D models of bone structure and soft tissues. After the DICOM image files were imported into the software, the images were segmented on the basis of gray intensities. Then, 3D reconstruction for each bone was attained using the CT Bone Segmentation procedure. The contours of the articular cartilages (femoral, tibial, and patellar), meniscus (medial and lateral), and ligaments (medial collateral (MCL), lateral collateral (LCL), anterior cruciate (ACL), posterior cruciate (PCL), and patellar tendon) were segmented from the MRI images. The manual segmentation process for bones and soft tissues was performed under the supervision of an experienced orthopedist and radiologist with an accuracy of 0.1 mm to minimize variation in the models. The assembled models are shown in Fig. 1a to c. Four locations of meniscus tears (anterior/posterior horn of medial/lateral menisci) were made using Magics 19.0 (Materialise, Leuven, Belgium) and is depicted in Fig. 1d. As shown in the figure, tears were modeled parallel to the edge at four horns of menisci, starting from the roots of the horns, with no extension to the body of both menisci. Four additional small longitudinal tears at four horns of menisci were also modeled with smaller defect at the horns compared to the previous tears (Fig. 1e).

### Finite element modeling and material properties

Data was exported as stereolithography (STL) files, and were imported to Materialise 3-matic 11.0 software (Materialise, Leuven, Belgium) to perform surface remesh. Considering that the ligaments are nonlinear materials, the quadratic hybrid formulation was used with a unit type of 10-node quadratic tetrahedron (C3D10H). For all other linear materials, the unit type was a four-node liner tetrahedron (C3D4).

The ligaments were modeled as transversely isotropic nearly incompressible neo-Hookean materials with the strain-energy function:

1 $$\Phi =C\_10\left({\left(G\_1\ \right)}^{-}-3\right)+\frac{1}{D_1}{\left(J\_F-1\right)}^2+S\left(\lambda \right)$$

Here, *S*(*λ*) denotes the strain energy function of the fiber family that satisfies the conditions:

2 $$\uplambda \frac{d S}{d\lambda}=\left\{\begin{array}{c}0,\lambda \le 1\\ {}{C}_3\left({e}^{\left(\lambda -1\right){C}_4}-1\right),1<\lambda <{\lambda}^{\ast }\ \\ {}{C}_5\lambda +{C}_6,\lambda \ge {\lambda}^{\ast }\ \end{array}\right.$$

*C*_10_ is a bulk material constant related to the shear modulus μ (*C*_10_ = 2/μ), *J*_*F*_ is the Jacobian of the deformation gradient *F*, and $\overline{G_1}$ represents the first invariant of the left Cauchy-Green tensor $\overline{G_1}= tr{\overline{FF}}^T$ with the modified deformation gradient $\overline{F}$ ($\overline{F}={J}_F^{-0.33}F$).

The stress in the fibers was dependent on the fiber stretch *λ*. The fiber stretch *λ* was determined from the deformed fiber orientation a_d_, the deformation gradient *F*, and the initial fiber orientation a_0_ (λ · a_d_ = *F* · a_0_). The fibers did not support any compressive stresses if they were under compression *λ* ≤ 1. The stiffness of the fibers increased exponentially when the fibers stretched between 1 and the pre-defined value (λ*). Beyond this stretch, the fibers straighten and the stiffness increases linearly. The constant *C*_3_ scaled the exponential stress, *C*_4_ was related to the rate of collagen uncramping, and *C*_5_ represented the elastic modulus of the straightened collagen fibers. The constant *C*_6_ was introduced to ensure stress continuation at λ* $\left[{C}_6=\left({e}^{C_4\left({\lambda}^{\ast }-1\right)}-1\right)\bullet {C}_3-\left({C}_5{\lambda}^{\ast}\right)\right]$. The material constants *C*_10_, *C*_3_, *C*_4_, *C*_5_, and *D*_1_ are listed in Additional file 1: Table S1 [14].

The bone material behavior was linear with an elastic modulus (*E*) of 7300 MPa and a Poisson’s ratio (ν) of 0.3 [15]. The articular cartilage and the menisci were assumed to be composed of a single-phase linear elastic and isotropic material with the following average properties: *E* = 15 MPa, ν = 0.475 and *E* = 120 MPa, ν = 0.45, respectively [16–19].

The number of nodes and elements of the intact knee model was shown in Additional file 1: Table S2. In order to reveal the exact stress on the meniscus, the edge length of meniscus was 0.5 mm, while that of bones, ligaments, and cartilages were 3.0 and 1.0 mm, respectively. Mesh convergence was tested for tetrahedral meshes of meniscus with element edge length from 1.0 to 0.2 mm. The results of the convergence study revealed that peak compressive stress was similar between the 1.0 and 0.5 mm conditions (average peak difference of 2.63%), while decreasing the element edge length from 0.5 to 0.2 mm did not cause any meaningful change, with more computation time considerably. For this reason, the element edge length of meniscus of 0.5 mm was selected for convergence. Finally, a total of 89,419 nodes and 383,145 elements were used in the finite element model of an intact knee.

### Loads and boundary conditions

The total tibiofemoral joint was incorporated for realistic knee joint characterization. In order to investigate the biomechanics of different postures, two FE simulations were applied: the static stance and slight flexion simulations. The boundary conditions were defined as follows: the tibia and fibula were fixed with full degrees of freedom at the lower end nodes, and the femur was unconstrained for all translational and rotational degrees of freedom. All ligaments were rigidly attached to their corresponding bones to simulate bone-ligament attachment. The kinematic constrain was modeled between femur and meniscus, meniscus and tibia, and femur and tibia for both the lateral and medial hemi joints. For static stance simulation, a vertical compressive load of 1150 N (two body weight) was applied on the femur at 0 degree of flexion, while a vertical compressive load of 1150 N and an anterior load of 350 N (60% of body weight) was applied on the top and anterior side of the femur at 0 degree of flexion for the slight flexion simulation, mimicking the force on knee joint during gait cycle in daily living activities [20, 21].

## Results

### Anterior horn tear of the meniscus

**Fig. 2:**
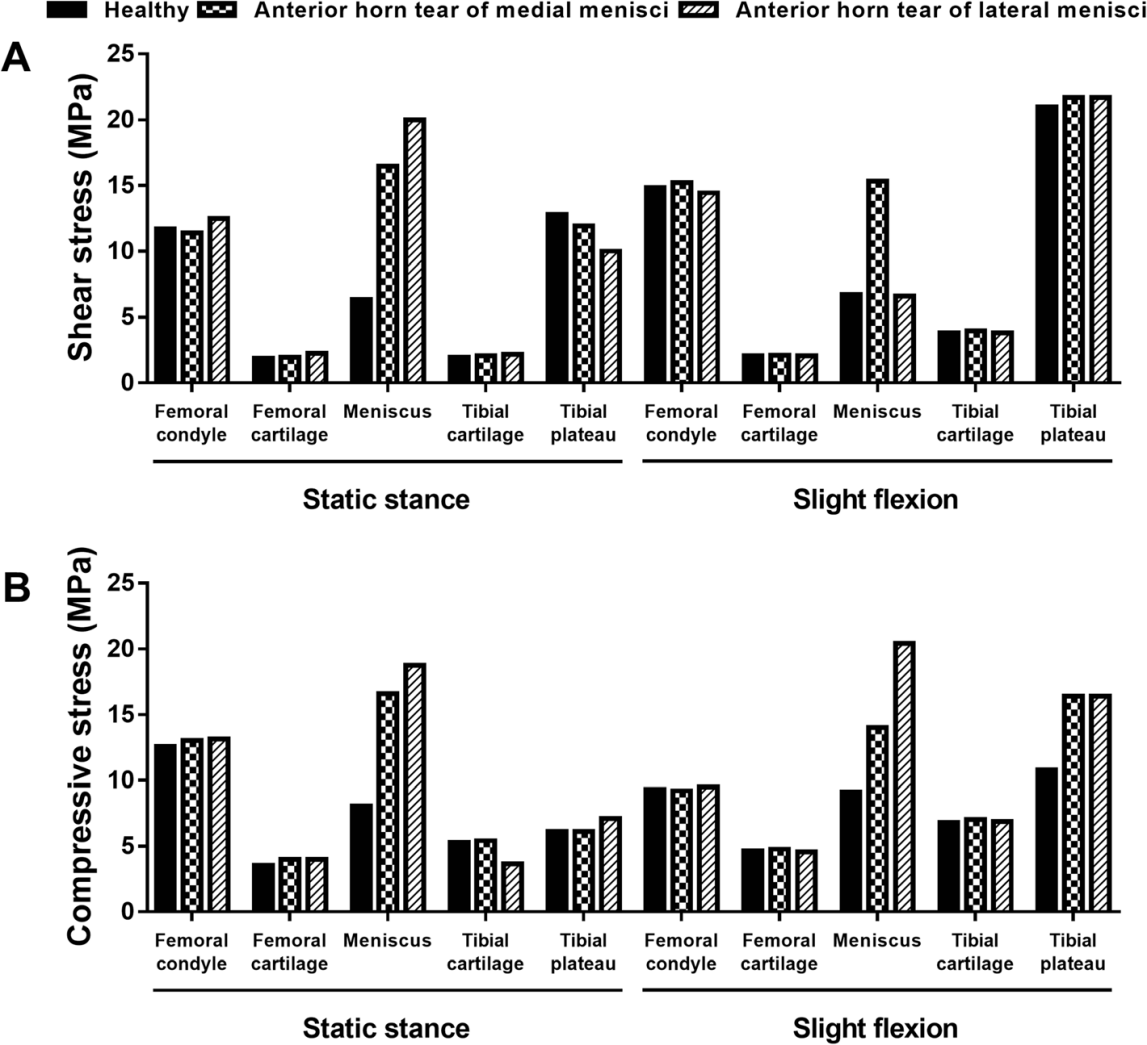
The maximum compressive and shear stress with longitudinal tears of anterior horns. **a** The maximum shear stress on the joint. **b** The maximum compressive stress on the joint

For the anterior tear of the medial meniscus, the stress distributions showed minimal changes (< 4%) on the subchondral bone of the femur, tibia, and femoral and tibial cartilage, for neither the static stance simulation nor slight flexion simulation. For the meniscus, the peak compressive (minimal principal) and shear (tresca) stress increased to 16.59 and 16.47 MPa under static stance simulation, and 14.01 and 15.34 MPa under slight flexion simulation (Fig. 2a).

With respect to the anterior tear of the lateral meniscus, similar minimal changes were found for the subchondral bone of the femur, tibia, and femoral and tibial cartilage, under either static stance simulation or slight flexion simulation. As to the meniscus, the peak compressive and shear stress increased to 18.76 and 19.99 MPa under static stance simulation, respectively, while 20.45 and 6.62 MPa under slight flexion simulation (Fig. 2b).

**Fig. 3:**
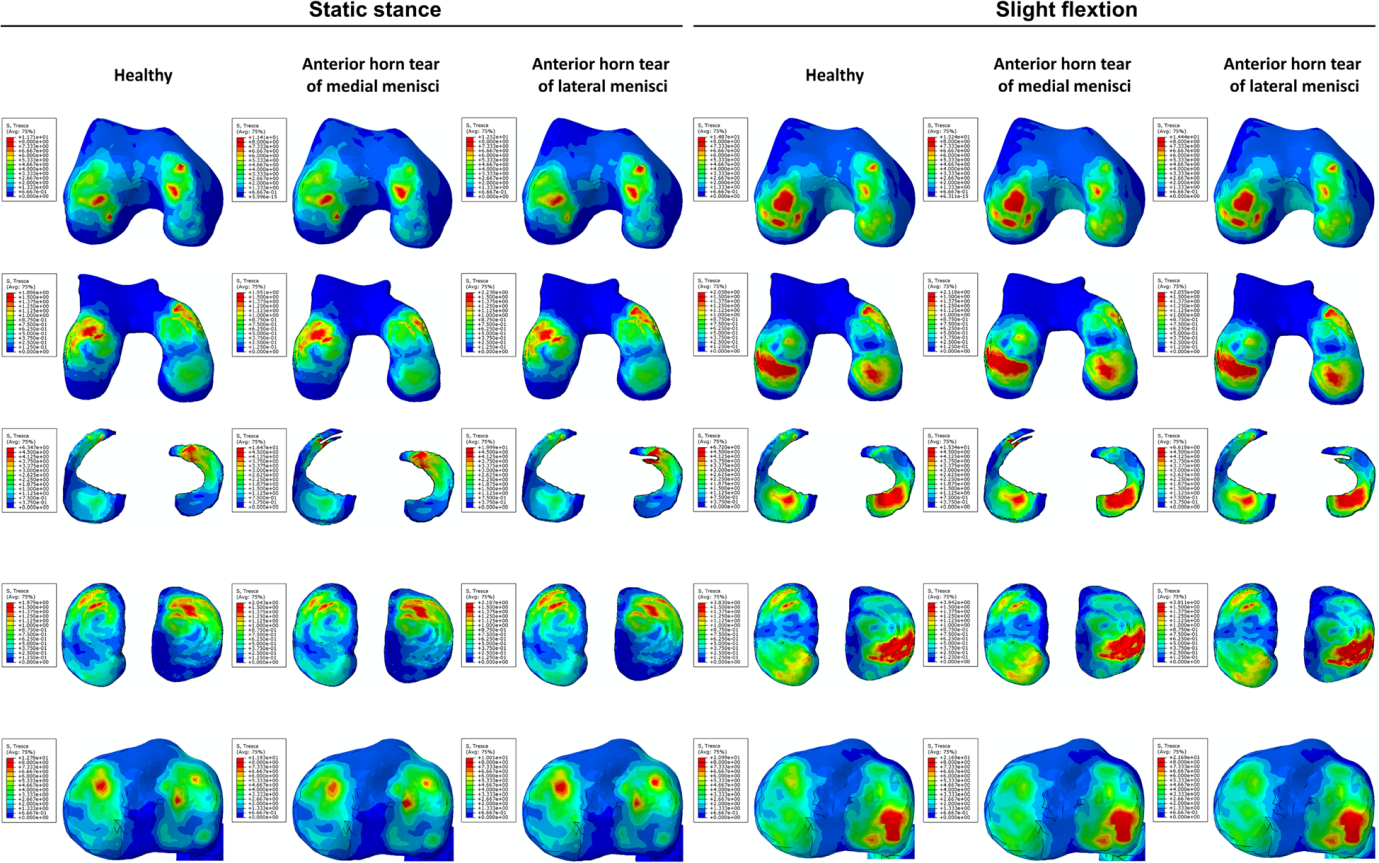
The results of shear stress on the joint with longitudinal tears of anterior horns. The results on the femoral condyles, femoral cartilage, menisci, tibial cartilage, and tibial plateau under static stance and slight flexion simulation were shown from top to bottom. Healthy subject and longitudinal tears of anterior horns were shown form left to right as the captions indicated

**Fig. 4:**
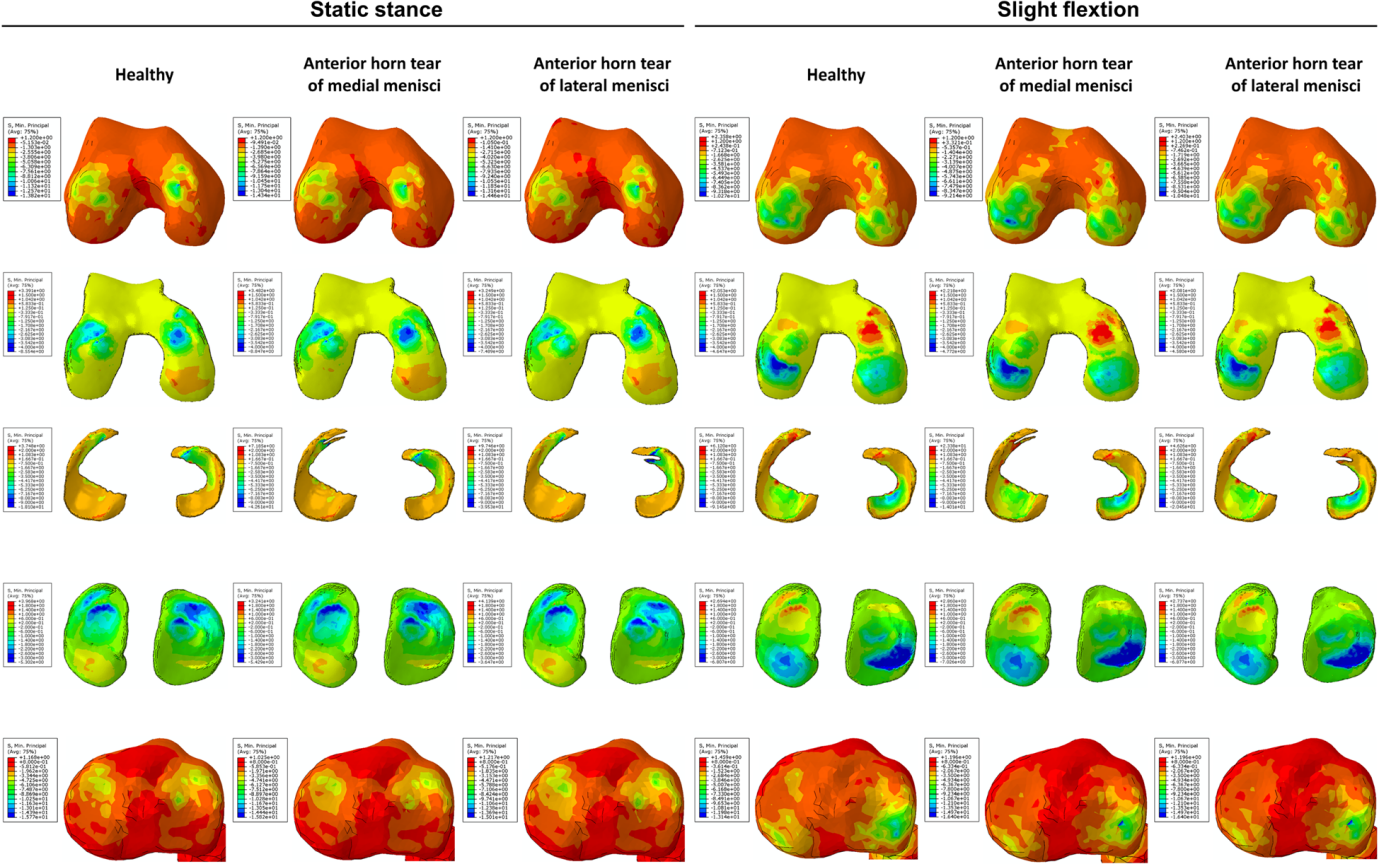
The results of compressive stress on the joint with longitudinal tears of anterior horns. The results on the femoral condyles, femoral cartilage, menisci, tibial cartilage, and tibial plateau under static stance and slight flexion simulation were shown from top to bottom. Healthy subject and longitudinal tears of anterior horns were shown form left to right as the captions indicated

From the nephogram for shear stress, it was observed that stress on the anterior part of the medial femoral condyle and cartilage was decreased while that on the anterior part of the lateral tibial plateau and cartilage was increased under both static stance and slight flexion simulation (when the longitudinal tear occurred at the anterior horn of the medial meniscus). The shear stress was more concentrated on the injured part of the medial meniscus under both simulations. For the longitudinal tear at the anterior horn of the lateral meniscus, no significant changes for shear stress distribution were observed when compared to no tear (Fig. 3). Similar results were found on the nephogram for compressive stress (Fig. 4). The compressive stress concentration altered from the medial to lateral of the femoral condyle under slight flexion simulation, while added stress was concentrated on the medial part of the femoral cartilage. Few changes were observed on the tibial plateau and cartilage under both simulations. For the meniscus, it was observed that increased compressive stress was concentrated on the tear location with the longitudinal tear on both horns of the meniscus under static stance simulation, while no significant changes were observed under slight flexion simulation.

The extrusion distances of the meniscus are shown in Table 1. The displacements of the medial and lateral meniscus were around 1.8 mm and 2.7 mm under static stance simulation, and 3.2 mm and 3.3 mm under slight flexion simulation. The displacements were significantly increased when the flexion simulation was applied on the knee and the longitudinal tear at the anterior medial meniscus and induced increased meniscus extrusion compared to the lateral meniscus injury.

**Table 1:** Meniscus extrusion under longitudinal tear at the anterior horn (mm)

Simulation	Static stance			Slight flexion		
Injury	Healthy	Medial	Lateral	Healthy	Medial	Lateral
Medial meniscus extrusion	1.80	1.81	1.92	3.157	3.31	3.22
Lateral meniscus extrusion	2.64	2.79	2.62	3.326	3.54	3.66

### Posterior horn tear of the meniscus

**Fig. 5:**
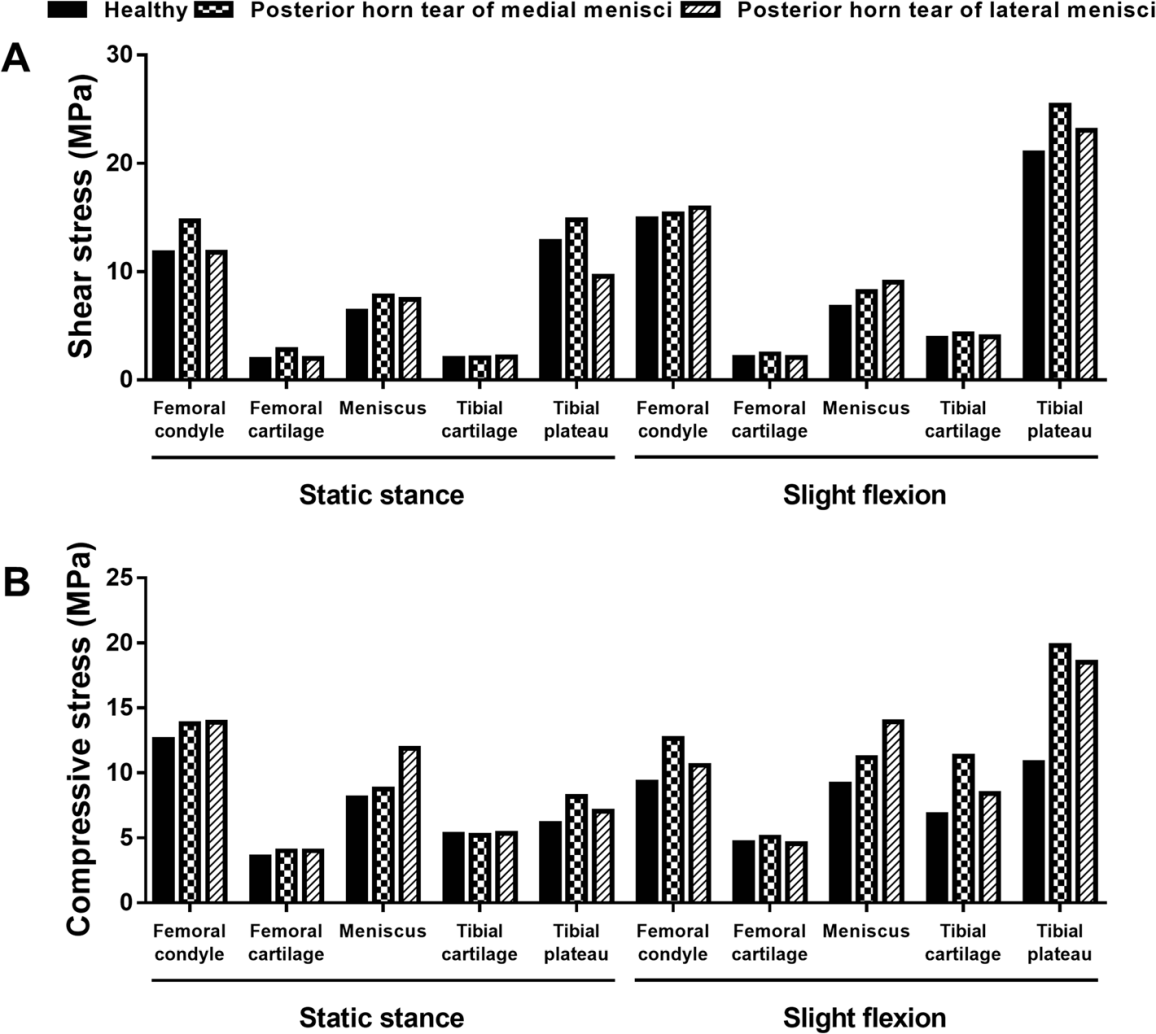
The maximum compressive and shear stress with longitudinal tears of posterior horns. **a** The maximum shear stress on the joint. **b** The maximum compressive stress on the joint

For the posterior tear of the medial meniscus, significant changes were found on the residual meniscus and femoral condyle. The peak compressive and shear stress of the meniscus increased to 8.77 and 7.79 MPa under static stance simulation and 11.18 and 8.16 MPa under slight flexion simulation, accounting for a 108%, 123%, 122%, and 121% increase respectively compared to no tear. For the femoral condyle, the peak compressive stress increased to 13.78 and 12.66 MPa and the shear stress increased to 14.66 and 15.34 MPa under static stance and slight flexion simulation, respectively (Fig. 5a).

With respect to the posterior tear of the lateral meniscus, only slight changes were observed on the subchondral bone and cartilage of the femur and tibia. For the meniscus, the peak compressive and shear stress increased to 11.89 and 7.44 MPa under static stance and 13.94 and 9.03 MPa under slight flexion simulation, respectively (Fig. 5b).

**Fig. 6:**
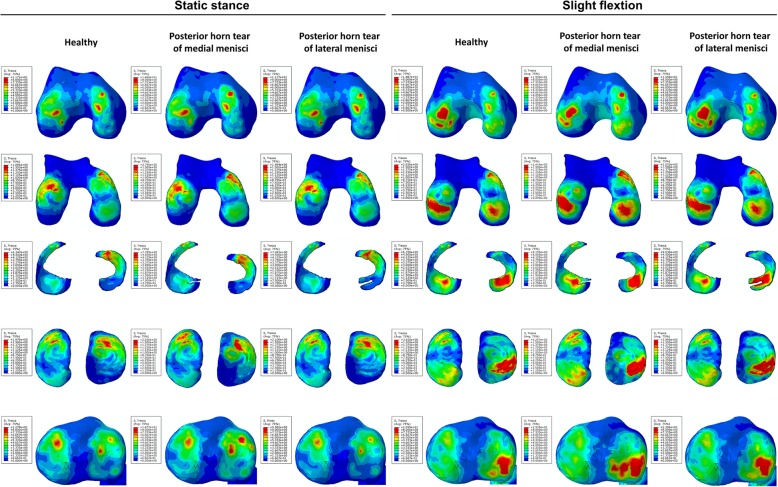
The results of shear stress on the joint with longitudinal tears of posterior horns. The results on the femoral condyles, femoral cartilage, menisci, tibial cartilage and tibial plateau under static stance, and slight flexion simulation were shown from top to bottom. Healthy subject and longitudinal tears of posterior horns were shown form left to right as the captions indicated

**Fig. 7:**
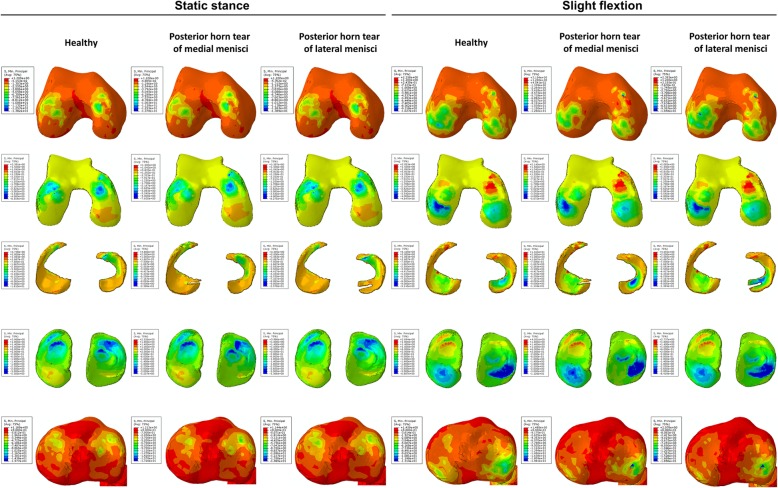
The results of compressive stress on the joint with longitudinal tears of posterior horns. The results on the femoral condyles, femoral cartilage, menisci, tibial cartilage, and tibial plateau under static stance and slight flexion simulation were shown from top to bottom. Healthy subject and longitudinal tears of posterior horns were shown form left to right as the captions indicated

Figure 6 shows that the medial femoral condyle and cartilage had increased shear stress compared to no tear, indicating increased shear stress transfer to the medial part. In particular, the shear stress was more concentrated on the medial femoral condyle under both simulations and was significantly higher compared to no tear and tear of the lateral meniscus. With regards to the tibial plateau and cartilage, increased shear stress was transversed onto the lateral part with longitudinal meniscus tears, especially with tears at the posterior horn of the medial meniscus. For the meniscus, increased shear stress was focused on the location of the tears and was observed under slight flexion simulation. With respect to the nephogram for compressive stress (Fig. 7), no significant changes were observed except for increased stress on the posterior horn of the lateral meniscus under slight flexion simulation.

Table 2 illustrates the extrusion distances. The meniscus was significantly extruded under both simulations when the longitudinal tear occurred at the posterior horn of the medial meniscus, compared to that of the lateral meniscus.

**Table 2:** Meniscus extrusion with longitudinal tear at the posterior horn (mm)

Simulation	Static stance			Slight flexion		
Injury	Healthy	Medial	Lateral	Healthy	Medial	Lateral
Medial meniscus extrusion	1.80	1.85	1.87	3.157	3.39	3.22
Lateral meniscus extrusion	2.64	3.02	2.79	3.326	3.89	3.35

## Discussion

The most significant finding of the present study was that longitudinal tears at different horns of the meniscus could cause diverse changes to the load distribution of the knee. Between all the four types of longitudinal tears investigated in this study, longitudinal tears at the posterior horn of the medial meniscus were found to be the most significant. The load on the subchondral bone and cartilage of the femur and tibia were significantly increased compared absence of tears.

We developed a detailed FE model of the knee joint with bones, cartilages, menisci, and main ligaments to evaluate the biomechanical changes after longitudinal tears at different horns of the meniscus. The results of compressive and shear stress of the healthy knee under static stance simulation were compared to previous studies (Table 3) [22–26]. The compressive stress on the menisci of our intact knee model was slightly higher than those of previous studies. However, the loads of studies were not the same and, as a result, the compressive stress remained different. Besides, the differences were not significant and were in the same order of magnitude. Therefore, we thought the results of stress from our FE simulation is in agreement with those studies and our FE model is validated to be used to explore the biomechanical effects of longitudinal tears.

**Table 3:** Comparison of the stress with published studies for the intact knee joint

	SSFC (MPa)	SSM (MPa)	SSTC (MPa)	CSFC (MPa)	CSM (MPa)	CSTC (MPa)
Our model	2.00	6.72	2.40	3.54	8.08	5.30
Shriram D et al.	1.93	–	2.32	2.76	–	3.52
E. Peña et al.	–	–	–	3.11	3.82	2.19
Verma et al.	–	–	–	–	–	5.96
Klets et al.	–	–	–	5.40	–	7.60
He et al.	–	–	–	3.34	6.52	–

*SSFC* maximum shear stress on femoral cartilages, *SSM* maximum shear stress on meniscus, *SSTC* maximum shear stress on tibial cartilages, *CSFC* maximum compressive stress on femoral cartilages, *CSM* maximum compressive stress on meniscus, *CSTC* maximum compressive stress on tibial cartilages

The meniscus is firmly attached to the tibial plateau by the anterior and posterior root and is critical for the proper biomechanical functioning of the meniscus. Clinically, treatment of meniscal tears include meniscectomy and meniscal repair. However, tears at roots of meniscus adjacent to the tibial insertion are extremely difficult to repair, as shortage of the meniscal substance left at the tibial insertion does not allow to secure firm stitches [27]. Meniscus tears can cause changes to the load distribution of the knee. The effect of tears to the anterior or posterior horn of the meniscus on load distribution for the knee components has not been investigated using FE models. Our study demonstrated that tears at the horns of the meniscus could induce alterations of load distribution on the meniscus or other knee components under both static stance and slight flexion simulations. Interestingly, tears at the anterior and posterior horn of the medial meniscus had more alterations of the biomechanics compared to the lateral meniscus, which suggests that the medial meniscus was more important than the lateral for load transmission and absorption to a certain extent. Allaire et al. studied the effect of a posterior root tear on the medial meniscus of the knee joint for contact pressure and kinematics and found that the tear would cause almost the same changes as total meniscectomy [28]. A biomechanical study conducted by Jin et al. found that the longitudinal tear at posterior horn of the medial meniscus significantly altered the knee kinematics, particularly the anterior-posterior tibial translation in the anterior cruciate ligament–deficient knee [29]. This suggests the important role of the medial meniscus posterior horn for knee biomechanics. The results of these studies revealed that a tear of medial meniscus posterior horn significantly change the load distribution of the knee, which is consistent with our findings compared to the other three types of tear. Table 4 showed the comparison of small longitudinal tears with previous large tears. Commonly, the smaller the size of tears, the larger the stress on menisci and the lesser the stress on tibial cartilage and plateau. It is reasonable as the meniscus become more intact, the meniscus would bear more stress of the knee. Under these circumstances, more stress of the knee was conducted to the meniscus and less was delivered to the tibial cartilage and plateau. When comparing the four small longitudinal tears, it was interesting that small tear at post horn of medial meniscus induced the least shear and compressive stress on the tibial cartilage and plateau, which also suggest the important role of medial meniscus posterior horn.

**Table 4:** Stress on knee components of small longitudinal tears compared to large tears (%)

Injured meniscus	Injured horn	Shear stress			Compressive stress		
		Menisci	Tibial cartilage	Tibial plateau	Menisci	Tibial cartilage	Tibial plateau
Medial	Front	188.70	78.41	98.12	258.89	90.24	91.27
	Post	176.48	71.14	78.55	351.90	73.29	79.67
Lateral	Front	178.34	75.28	93.92	122.08	89.82	85.04
	Post	153.81	81.11	82.79	165.02	87.92	91.35

Patients with meniscal root tears often complain of only minimal mechanical symptoms or discomfort with full flexion [30]. However, there has been growing concern of meniscal root tears because of the important physiological role played by the meniscal root as well as the advancement in various diagnostic tools. Many factors affect the choice of treatment of meniscal root tears, including the severity of the injury, timing of injury to surgical intervention, and the condition of the articular cartilage. Commonly, elderly patients with high grade OA (Outerbridge 3–4) are usually candidates for nonoperative treatment. Use of analgesics and activity restriction are recommended to relieve the symptoms [31]. Patients with advanced degenerative changes, persistent mechanical symptoms, and who have failed conservative treatment may benefit from a partial or subtotal meniscectomy, especially for those aged more than 45. Surgical repair of meniscal root tears is recommended to prevent the damage toward meniscus and osteoarthritis, except for those who cannot bear the surgery, with diffuse Outerbridge grade 3 or 4 OA of the ipsilateral compartment, non-symptomatic chronic meniscal root tears, and/or significant limb malalignment unless concurrently corrected [32]. Our results showed significant change of biomechanics of the joint after medial meniscal posterior root tear, which reminds the surgeons to pay more attention to the diagnosis of such tears and to resume the normal distribution of biochemical loads by appropriate treatments.

**Fig. 8:**
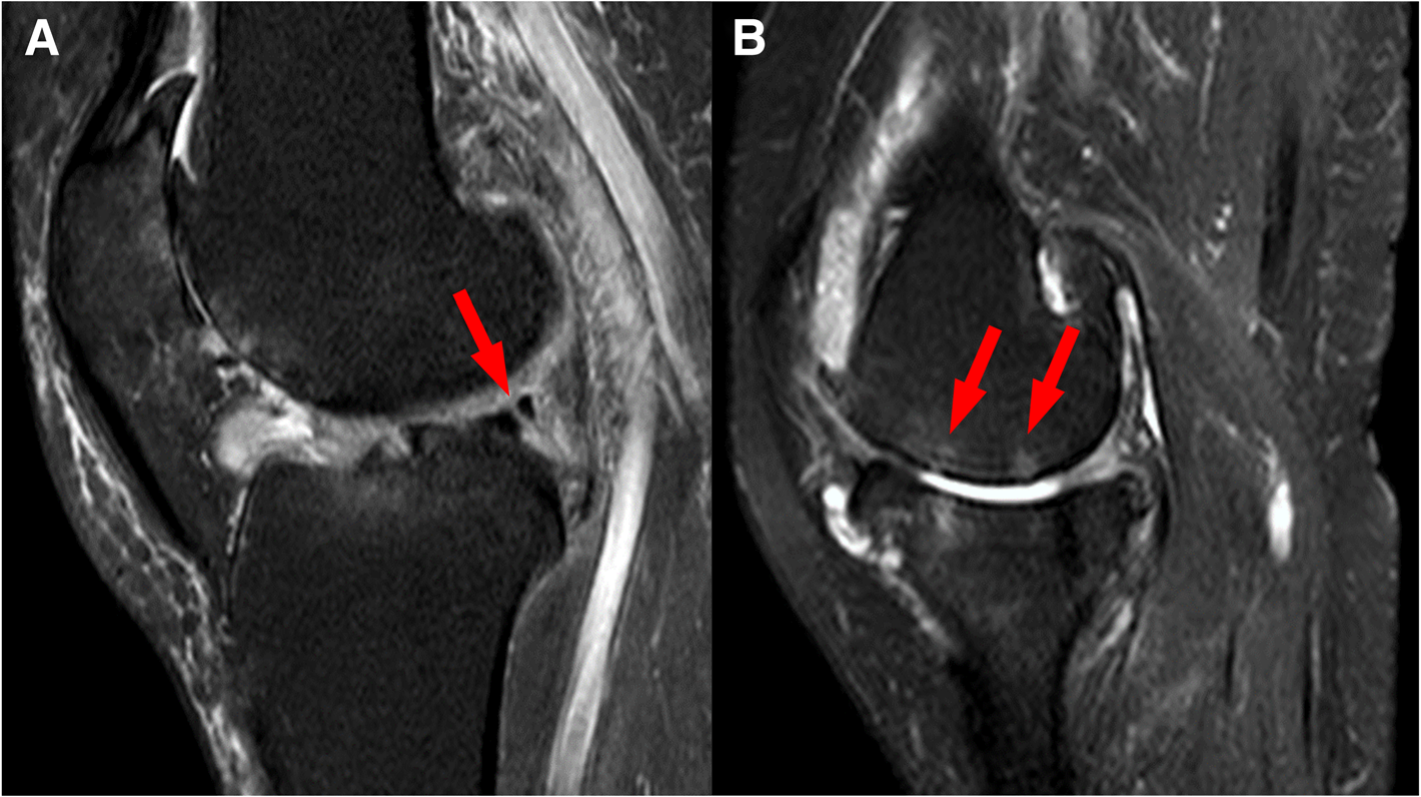
The MRI results of a 49-year-old female patient. **a** Red arrow indicates the longitudinal tear of posterior horn of medial meniscus. **b** Red arrows show the cystic degeneration and subchondral bone osteonecrosis. The cartilage damage can also be observed

Elevated load stress on the subchondral bone was thought to be one of the risk factors for osteonecrosis [33]. Previous studies have demonstrated the association between the tear of the medial meniscus posterior horn and spontaneous osteonecrosis of the subchondral bone of the femoral condyle. Robertson et al. identified 30 patients with spontaneous osteonecrosis of the medial femoral condyle, and found that 80% of these patients had tears in the posterior root of the medial meniscus [34]. Muscolo et al. also reported five patients with medial meniscus degenerative tears and found comparable osteonecrosis of the femoral condyle after a duration of 2.7 months [6]. In this study, we also found similar cases with medial meniscal tears and spontaneous osteonecrosis (Fig. 8). We noticed higher compressive and shear stress on the medial femoral condyle with longitudinal tear at the posterior horn of the medial meniscus compared to other tears. The load on the bone exceeding 20 MPa could induce microfractures, which results in osteonecrosis [35]. Although the precise mechanism between tears of the medial meniscus posterior horn and spontaneous osteonecrosis of the subchondral bone of the medial femoral condyle remains to be elucidated, several hypotheses have been proposed. One of them is the meniscal extrusion theory. It is evident that the meniscus would extrude outwards resulting in increased contact area between the femoral condyle and tibial plateau. This increases the mechanical load between the bones to induce osteonecrosis [36]. Meniscal extrusion was investigated in our study. Our results showed significantly more medial and lateral meniscus extrusion with tears of the medial meniscus posterior horn under slight flexion simulation. This is consistent with the meniscal extrusion hypothesis and may provide some guidance for understanding the mechanism between tears of the medial meniscus posterior horn and spontaneous osteonecrosis of the subchondral bone of the medial femoral condyle.

The present study has several limitations. Firstly, all loads were applied at almost full extension of the knee. More results would be obtained with different flexion angles of joint. Secondly, periarticular soft tissues, such as capsule and other ligaments, were found to restrict the extrusion of meniscus. This was ignored in our model. Thirdly, the sample size was small in this study. Thus, it is difficult to perform statistical analysis on the results. Despite the limitations, the strength of our study is that we revealed the stress transforming on subchondral bone in the post-injured knee, apart from the traditionally studied cartilages and menisci. Young’s modulus of bone is much higher than that of the connective tissues. Thus, previous studies assumed the bone structures as rigid body and ignored the stress concentrated on the bone, which could explain the mechanism of subchondral bone osteonecrosis [10, 22, 37].

## Conclusion

In this study, a detailed 3D knee FE model was developed. The effect of longitudinal tears at the different horns of both medial and lateral meniscus of the knee joint, and associations between meniscal horn tears and spontaneous osteonecrosis of the subchondral bone were studied. This study permits for a better understanding of the important role of the meniscus in load transmission and helps to investigate the mechanism between tears of the medial meniscus posterior horn and spontaneous osteonecrosis of the subchondral bone.

## Additional file

Additional file 1: **Table S1.** Material constants for the ligaments. **Table S2.** Number of nodes and elements of the intact knee model. (DOCX 17 kb)

## Data Availability

Please contact author for data requests.

## Abbreviations

ACL: Anterior cruciate ligament
CSFC: Maximum compressive stress on femoral cartilages
CSM: Maximum compressive stress on meniscus
CSTC: Maximum compressive stress on tibial cartilages
FE: Finite element
FOV: Field of view
LCL: Lateral collateral ligament
MCL: Medial collateral ligament
PCL: Posterior cruciate ligament
SSFC: Maximum shear stress on femoral cartilages
SSM: Maximum shear stress on meniscus
SSTC: Maximum shear stress on tibial cartilages
