# Supplementary material for: The biomechanical changes of load distribution with longitudinal tears of meniscal horns on knee joint: a finite element analysis
Source: J Orthop Surg Res. 2019 Jul 25;14:237. doi: 10.1186/s13018-019-1255-1 (PMC6659249; doi:10.1186/s13018-019-1255-1)
Supplement: Supplementary file 1 — Table S1. Material constants for the ligaments. Table S2. Number of nodes and elements of the intact knee model. (DOCX 17 kb) [file 13018_2019_1255_MOESM1_ESM.docx]

**Additional file 1**

**Table S1.** Material constants for the ligaments

|  | C_10_ (MPa) | C_3_ (MPa) | C_4_ (-) | C_5_ (MPa) | D_1_ (MPa^-1^) | λ* (-) |
| --- | --- | --- | --- | --- | --- | --- |
| ACL | 1.95 | 0.0139 | 116.22 | 535.039 | 0.00683 | 1.046 |
| PCL | 3.25 | 0.1196 | 87.178 | 431.063 | 0.0041 | 1.035 |
| LCL | 1.44 | 0.57 | 48.0 | 467.1 | 0.00126 | 1.063 |
| MCL | 1.44 | 0.57 | 48.0 | 467.1 | 0.00126 | 1.063 |
| PT | 3.25 | 0.1196 | 87.178 | 431.063 | 0.0041 | 1.035 |

ACL: Anterior cruciate ligament; PCL: Posterior cruciate ligament; LCL: Lateral collateral ligament; MCL: Medial collateral ligament; PT: Patellar tendon

**Table S2.** Number of nodes and elements of the intact knee model

|  | Nodes | Elements |
| --- | --- | --- |
| Meniscus | 9597 | 40411 |
| ACL | 1286 | 5535 |
| PCL | 2005 | 8948 |
| LCL | 2474 | 10279 |
| MCL | 2110 | 7522 |
| PT | 4893 | 16484 |
| FC | 20305 | 82929 |
| TC | 11710 | 46517 |
| PC | 7055 | 28116 |
| Femur | 14122 | 70430 |
| Tibia | 12379 | 58940 |
| Patella | 1483 | 7034 |

ACL: Anterior cruciate ligament; PCL: Posterior cruciate ligament; LCL: Lateral collateral ligament; MCL: Medial collateral ligament; PT: Patellar tendon; FC: Femoral cartilage; TC: Tibial cartilage; PC: Patellar cartilage
